# Development of a flexible, sweat-based neuropeptide Y detection platform†

**DOI:** 10.1039/d0ra03729j

**Published:** 2020-06-17

**Authors:** Nathan Kodjo Mintah Churcher, Sayali Upasham, Paul Rice, Serena Bhadsavle, Shalini Prasad

**Affiliations:** Department of Bioengineering, University of Texas at Dallas Richardson TX-75080 USA Shalini.Prasad@utdallas.edu

## Abstract

Neuropeptide Y (NPY) biomarker levels have a close association with the diagnosis of Major Depression Disorder (MDD) and anxiety disorders. Quantifying NPY in correlation to self-reported symptoms will be an important measure to ensure a relatively uniform diagnosis and help with disease prognosis of these disorders. The work presented is a novel, passive eccrine sweat based, electrochemical detection platform for quantification of NPY biomarker levels. The paper offers a comparison between non-porous and porous sensor platforms using various electrochemical detection techniques. This work uses a novel strategy towards designing an optimal nanobioelectronic interface to measure NPY. The portability aspect of this detection platform is discussed by the demonstration a novel, portable EmStat Pico based electronic platform. The detection limit of the sensor is 10 pg mL^−1^ and its range is 20–500 pg mL^−1^. The NPY detection platform is envisioned to be a wearable point-of need monitoring system for management of chronic anxiety disorders and MDD.

## Abstract 2

Novel wearable NPY biomarker tracking system, envisioned to be a chronic anxiety and MDD management platform *via* self-monitoring.

## Graphical abstract

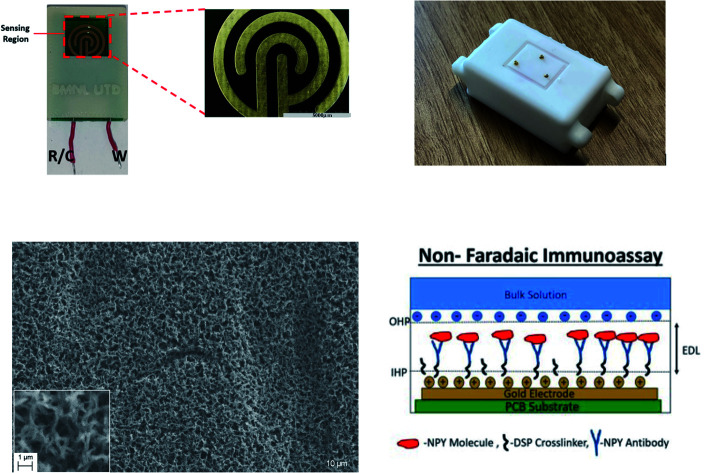

## 1 Introduction

Clinical depression, medically known as Major Depression Disorder (MDD) is the most common mental disorder characterized by symptoms like overwhelming feeling of sadness, isolation, and despair that usually lasts for at least two weeks at a time. According to the World Health Organization's (WHO) global health estimates (2015), it was reported that 4% of the global population suffers from depression. Anxiety disorders are another contributor to these statistics as it is estimated that 3.6% of the global population struggles with anxiety disorders.^1^ In the United States, according to the statistics determined by the National Institute of Mental Health (NIMH), as of 2017, 17.3 million adults have had at least one MDD episode.^2^ Out of this population group, 3.4% succumb to suicide and an estimated 60% of those people were known to be suffering from some form of depression or anxiety disorder. Currently, there are no quantifiable biological tests to confirm the diagnosis of MDD. Apart from the variability in diagnosis of MDD due to the different phenotypes presented by the disorder, diagnosis is solely based on the clinical judgement and experience of doctors which adds another factor to this gap in disease identification. Quantifying a biomarker in correlation to self-reported symptoms of MDD will be an important measure to ensure a relatively uniform diagnosis of MDD across the globe as well as help in the monitoring and treating patients already diagnosed with the disorder. Neuropeptide Y (NPY) has been identified as such a biomarker and has been shown to have a significant increase in concentration in patients diagnosed with MDD.

Neuropeptide Y (NPY) is a neuropeptide belonging to the pancreatic polypeptide family involved in various essential biological processes such as stress, cardiovascular regulation, appetite, and neuroendocrine modulation.^3^ It is about 36 amino acids long and one of the most abundant neuropeptides in the central nervous system, synthesized in the arcuate nucleus (ARC) of the hypothalamus.^4^ A proper functioning NPY system has anxiolytic effects, partially counteracting some of the physiological effects and hormones associated with an elevated stress level. High levels of NPY has been shown to protect against the development of disorders such as PTSD and bipolar disorder, whereas depressed levels can lead to a higher risk of chronic stress and stress-related disorders;^5^ thus, NPY is a good molecule to study to help quantify a patient's level of stress and their body's ability to counteract these stress levels. Currently, these mental disorders often go undiagnosed due to their subjective nature. A biosensor solution such as the one described in this paper offers discrete, objective, and actionable data for a more accurate diagnosis prospect. Current NPY biosensor solutions (highlighted in Table 1), while capable of NPY detection within physiological ranges, often are invasive or may lack sensitivity for the detection of NPY that is necessary for prognostic monitoring. Sampling for the analyte required to measure NPY levels would typically involve pricking or drawing blood. If the purpose of measuring the biomarker is to diagnose the effect of stress on the body or to quantify anxiety, then the stress created during sampling might add to the response and give a false sense of biomarker elevation. Commercially, biomarker estimation is performed using Enzyme Linked Immunosorbent assays (ELISAs) or Radioimmunoassays (RIAs). These techniques are sensitive but require large sample volumes and results are not in real-time. For monitoring stress in the body, we need a system that would measure the biomarker levels in real time as stressful events or episodes are often time sensitive. Sweat based biosensing offers a potential solution to this problem.^6^ Not only does it offer a non-invasive path for biomarker estimation, but it also facilitates usage of lower sample volumes for detection. For point-of-care and point-of-need devices, sweat based biosensing is a very important bridge to cross. It provides significant improvement in performance, comfort, functionality, and cost effectivity of healthcare diagnostic and monitoring systems.^7^ This technology is sensitive, rapid and can be made reusable. When coupled with electrochemical transducers, these sweat based sensors can be used for combinatorial detection of multiple biomarkers and can be miniaturized and mass-produced.^8^

**Table 1:** Current NPY biosensor research

Description	Type of sensor	Biofluid	Limit of detection	Reference
Label-free photonic guided mode resonance (GMR) biosensor, operating in near-IR wavelengths	Optical	PBS at pH = 7.4	Limit of detection of 0.1 pM with a detection range of 0.1–10 pM	9
Quartz substrate with a glass cover slip microfabricated with electrochemical working, counter, and reference electrodes	Electrochemical	Cerebrospinal fluid, sweat, saliva, and blood serum samples	Limit of detection of 4 pM	10
Single-walled carbon nanotube (SWCNT) – aptamer biosensor with a label-free multisensing platform	Electrochemical	Blood serum	Limit of detection of 500 pM with a detection range of 1 μM to 100 pM	11
Aptamer and gold nanoparticle (AuNP) colorimetric sensor	Colorimetric	Artificial sweat	Limit of detection of 110 nM with a detection range of 50–400 nM	12
Sensing platform based on aptamer-immobilized graphene-gold nanocomposite microelectrodes embossed on cyclic olefin copolymer	Electrochemical	Blood serum	Limit of detection of 10 pM with a detection range of 10–1000 pM	13
Gold microelectrode based wearable NPY detection and monitoring platform	Electrochemical	Sweat	Limit of detection of 10 pg mL^−1^ with a range 10–500 pg mL^−1^	This work

These can be used as a self-monitoring platform for tracking user well-being; however, there are some hurdles on the path to perform successful NPY detection. For example, the physiological range of NPY in sweat is 50–200 pg mL^−1^. This poses a challenge as the detection system needs to be robust enough to generate a signal for concentrations that are a magnitude lower than those in commercially used gold standard biofluids like blood or serum. In a wearable system, electrode flexibility is an imperative. A very interesting approach has been adopted by B. Gao *et al.*, where they have created a highly stretchable and flexible artificial skin platform inspired by fish and reptile scales. This “Kirigami”, a subset of origami artwork, patterns paper to mimic microfluidic and electronic devices used to perform biomarker detection.^14^ There is usually a tradeoff between conformation to the user's body movement and being mechanically resilient so that the signal is not degraded by motion artifacts. In this work, we aim to address these issues with a novel, flexible sweat NPY detection platform.

This work highlights a sensor prototype for a non-faradaic electrochemical biosensor for the detection of NPY within human sweat. It offers the advantages of being cost-effective, non-invasive, label-free, rapid, and sensitive. By offering a comprehensive analysis of porous *vs.* non-porous membranes, this paper highlights the importance of substrate optimization while designing wearable sensing technologies. Binding studies using FTIR confirm the functionality of the capture probe used in this system. Evaluation of the performance of both systems *i.e.* non-porous and porous systems was carried out using Electrochemical Impedance Spectroscopy (EIS). By using this detection modality, detection becomes rapid and label free, which is ideal for a point of need diagnostic and self-monitoring device. An alternative technique, chronoamperometry, confirms the sensitivity of the sensing platform to various NPY concentrations. We also discuss the usability of the system as a benchtop device *vs.* a wearable using a novel portable device which utilizes the commercially available EmStat Pico from Palmsens. The specificity of the sensor in the presence of interferents in sweat is also confirmed. Thus, this convenient biosensor platform functions using ultralow volumes of sweat, an easily accessible analyte, and addresses the need for a self-monitoring device for quantification of stress.

## 2 Results and discussion

### 2.1 Sensor platform characterization

We have evaluated two types of sensing platforms for NPY detection one with a non-porous substrate and another with nano-porosity. We have leveraged our findings from the non-porous substrate and translated it to the nanoporous substrate which is more suitable for wearable bio sensing applications.

#### 2.1.1 Non-porous electrode design characterization

**Fig. 1:**
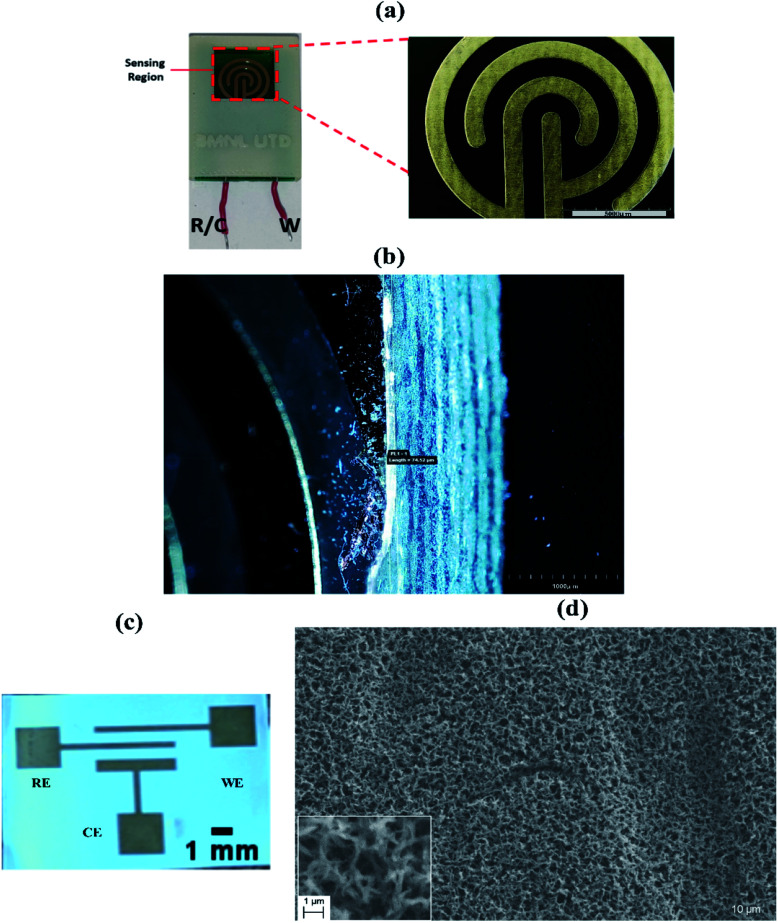
(a) Non-porous sensing platform describing reference/counter (R/C) and working (W) electrode design (left), circular gold microelectrode design (right), (b) microscopic capture of circular gold electrode highlighting the electrode structure, (c) porous electrode design, three electrode interdigitated system with working, reference and counter electrodes, and (d) scanning electron micrograph to highlight the nanoporosity of the substrate.

The basis for the use of a non-porous substrate was to provide a rapid, relatively low volume, benchtop alternative to the current methods of detection of NPY in sweat. The non-porous platform is a two-electrode system (counter and reference electrode combined as one and a working electrode) made up of a gold microelectrode electroplated on a FR-4 printed circuit board as shown in Fig. 1a. This figure also shows the concentric interdigitated electrode pattern with an interspacing distance of 1 mm chosen to minimize field interferences with signal measurements due to the presence of edges.^15^ The total surface area of electrode is 135 mm^2^ with the ratio of the surface area of the working electrode to the reference/counter electrode to be around 1 : 10. The design of the gold electrodes microstructure provides a good anchoring platform for the immobilization of the self-assembled monolayer (SAM) for the detection of NPY in sweat. Also, this non-porous platform acts as optimal base substrate which helps in preliminary assay optimization studies. This is because non-porous platforms do not have the variability from variations in binding interactions created due to effective surface area coverage during fluid confinement inside the porous structure. Fig. 1a and b highlight the thickness of the gold electrodes that were used for electrochemical measurements. The SAM is formed on the biosensor illustrated by the schematic in Fig. 3c. The functionalization is described in detail in Section 2.2. In this work, we characterized and optimized the binding interactions between NPY and the immobilized detection probe using the non-porous substrate. After determining the sensing response and optimizing for the detection assay, the electrode substrate was switched to a porous platform to tune it towards a wearable application.

#### 2.1.2 Nano-porous electrode design characterization

Nano-porous membranes have been widely used as substrates for sweat based detection.^16–18^ Porous membranes offer integrated wicking for sweat sampling. In typical passive sweat dependent devices, the sweat volume collected averages between 5–10 μL, which is based on average sweating of 3–5 nL per min per gland.^19^ This work uses ultralow volumes of 5 μL of sweat samples which facilitates easy sampling conditions, making it ideal for self-monitoring. The electrode is a three-electrode interdigitated electrode system. It has been illustrated in Fig. 1b. This electrode design increases the overall capacitance of the system and is designed to operate with ultra-low volumes of analyte. This gold electrode is then functionalized using a thiolated SAM layer. The details about fabrication and functionalization are described in the Experimental section. The substrate used is in this electrode system is a nanoporous polyamide membrane. A Scanning Electron Micrograph (SEM) is presented in Fig. 1b. The image highlights the crosslinked porous structure of the membrane. Porous systems have significant advantages over flexible non-porous substrates. Nanoporous substrates are an attractive substrate for many biomedical applications. Biosensing with these membranes typically results in high sensitivity because of their integrated crosslinked structure that offers optimal fluid transport. Some of the key phenomena involved in increasing the efficiency of biosensing performance by using nanoporous membranes are macromolecular crowding,^20^ nanoconfinement,^21^ and size-based exclusion.^22^ A schematic of these processes has been illustrated in Fig. 4a and b. This is further described in Section 2.4.

### 2.2 Immunochemistry characterization

**Fig. 2:**
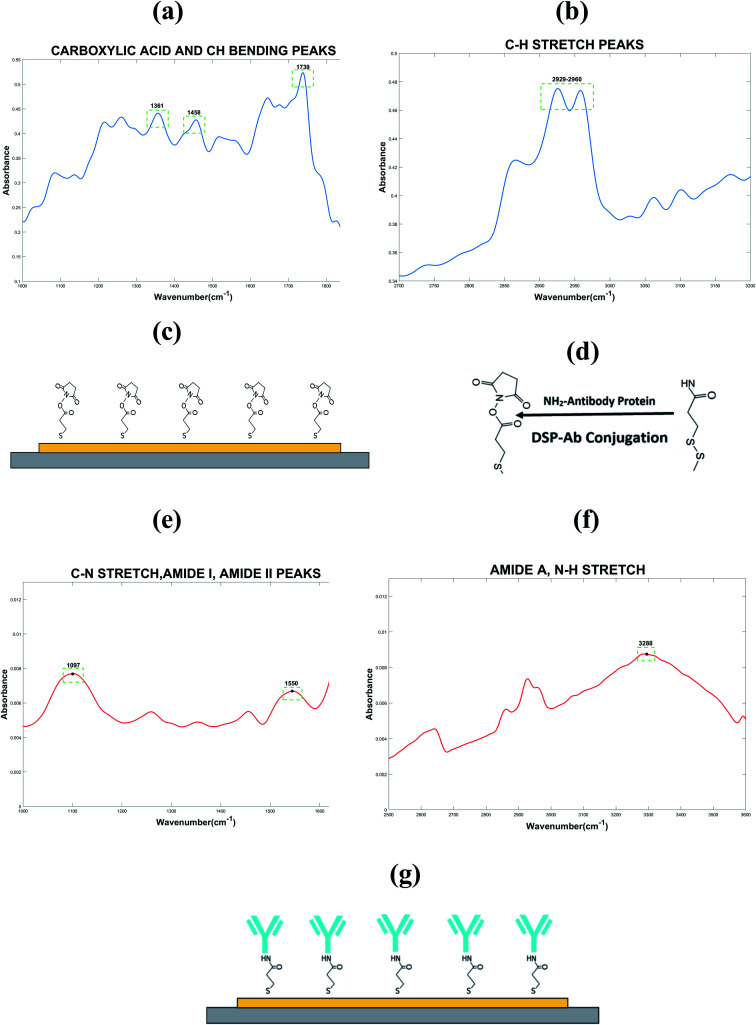
FTIR spectrum for thiol linker, DSP describing the (a) carboxylic acid, C–H bending peaks and (b) C–H stretch peaks, (c) schematic highlighting thiol–gold chemistry used for functionalization, (d) chemistry for linker and antibody binding; FTIR spectrum for capture probe highlighting (e) amide I, II peaks and (f) amide A, N–H stretch, and (g) schematic illustrating the binding of capture probe-antibody (blue) to the linker for immobilization on sensor surface.

Validation of the formation of SAM on the biosensor electrode surface was done by Fourier-Transform Infrared Spectroscopy (FTIR). The spectrum for thiol linker, dithiobis-[succinimidyl propionate] (DSP) is depicted in Fig. 2a and b. FTIR was used to first validate the successful chemisorption of the cross-linker onto the gold electrode surface as illustrated in the schematic shown in Fig. 2c. It was also used to provide a binding site as a means of anchoring the NPY-antibody to the thiolated gold surface. This will act as the detection probe for this sensing platform as illustrated in the schematic shown in Fig. 2d and e. Table 2 presents the relevant IR spectra observed for the linker and antibody immobilized onto the gold electrode surface extracted from the FTIR spectrum graph shown in Fig. 2a, b and e, f respectively.

**Table 2:** FTIR peak confirming SAM immobilization

Description	Expected peak position (cm^−1^)	Observed peak position (cm^−1^)
Stretching of CH alkane chain	2640–3000	2949
Free carboxylic acid	1743	1739
CH_2_ bending	1465	1458
CH_3_ bending	1375	1361
Amide-I bond	1600–1700	1657
Amide-II bond	1510–1580	1550
C–N stretch	1020–1250	1097
Amide A, N–H stretch	3225–3280	3288

The peak observed at 1739 cm^−1^ is a characteristic peak associated with the binding of DSP binding to gold, which indicates the presence of free carboxyl acid and the asymmetric stretching of the *N*-hydroxysuccinimide (NHS) ester. The peak observed at 2949 cm^−1^ is as a result of the stretching of the alkane chain present in DSP. Peaks also observed at 1458 cm^−1^ and 1361 cm^−1^ indicate the vibrations of methylene scissors in DSP and the symmetric stretching of C–N–C stretch of NHS. These observed peaks validate the formation of a SAM of DSP confirming the chemisorption of DSP to the gold surface. Upon the DSP–antibody, there is a visible reduction of the peak observed at 1739 cm^−1^ indicative of the breaking of the CO–NHS bond in the DSP to allow for the reaction with the primary amine groups of the antibody to form stable amide bonds. The peaks observed at 1657 cm^−1^ and 1550 cm^−1^ are indicative of the absorbance peaks associated with the formation of amide I and amide II bonds, respectively. The peak observed at 1097 cm^−1^ and 3288 cm^−1^ correspond to the C–N stretch and N–H stretch in the formation of the SAM capture probe on the gold electrode surface.^23,24^

### 2.3 Sensor performance in non-porous substrate

#### 2.3.1 Sensitivity using a rigid non-porous substrate

Gold has been used for many decades for the design of biosensors because it is biocompatible and it has desirable electrical and optical properties. In this paper, we make use of the electrochemical stability and biocompatibility of gold to create a sensitive immobilization platform with the ability to characterize the changes in the electrical characteristics at the electrode–electrolyte surface in the presence of NPY in sweat. The sensor platform consists of a gold micro-electrode electroplated on to a FR-4 printed circuit board. The design dimensions of the electrodes were chosen to increase the surface area of the sensor to ensure effective binding of the cross-linker and NPY antibody to build the platform for the detection. This in-turn increases the capacitance in the electrical double layer which is observed in a non-faradaic EIS measurement for characterization of the formation of an antibody–antigen complex formed on the sensor surface.

#### 2.3.2 Sensor detection response using electrochemical impedance spectroscopy (EIS)

**Fig. 3:**
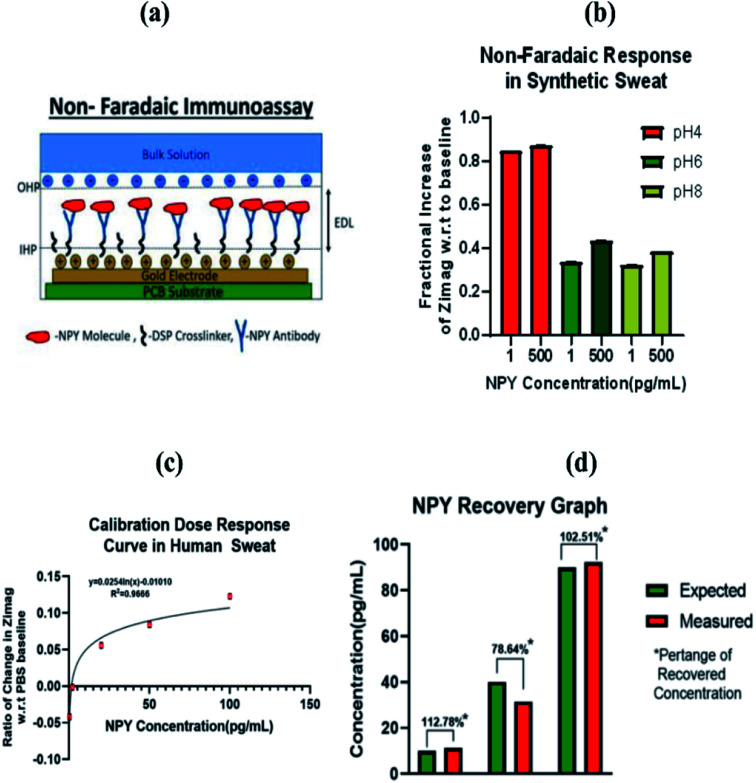
(a) Schematic illustrating Electric Double Layer (EDL) in a non-faradaic immunoassay, OHP-Outer Helmholtz Plane, IHP-Inner Helmholtz Plane, (b) non-faradaic EIS response of non-porous sensor system in synthetic sweat with varying pH, (c) calibrated dose response for NPY detection in human sweat, and (d) Comparison of measured *vs.* recovered concentration for sensing system.

Characterizing the sensor's immunoassay on a rigid non-porous substrate is important because it provides useful information about the interactions between the electrode and the biofluid. Additionally, it offers us an insight into parameters to optimize prior to transitioning to a flexible non-porous substrate, which is more adaptable to a wearable form factor, point of care device (POC). In this characterization we employed the use of an AC label-free technique known as non-faradaic electrochemical impedance spectroscopy. When a biofluid, in this case sweat, is introduced onto the biased gold electrode, the interactions between the ions in the sweat, including the target molecule, NPY, and the immobilized self-assembled monolayer will contribute towards the signal response. The gold electrode surface is positively charged when biased and its interactions with charged solution ions present in sweat gives rise to an Electrical Double Layer (EDL), a model used to characterize the interactions that occur at the electrode–electrolyte surface first proposed by Helmholtz in the 1850's.^25,26^ There have been further modifications and additions to the model first proposed by Helmholtz; however, the fundamental notion of the formation of a double layer when an electrolyte encounters an electrode still stands. The positively charged gold electrode attracts negative ions to approach the electrode surface to form a layer in order to balance out the positive charge on the electrode surface. This leads to the formation of two electrically charged layers with the immobilized SAM formed on the electrode surface. This is illustrated clearly in the schematic shown in Fig. 3a, clearly showing the two layers, Outer Helmholtz Layer (OHL) or plane and Inner Helmholtz Layer/plane (IHL) which are modelled as the two charged plates of a capacitor. The SAM formed lies between the two layers contributes as a dielectric of the capacitive system denoted as the EDL in the non-faradaic system. Capacitance is directly proportional to the permittivity of the dielectric which are dependent on the NPY binding activity, which in turn modulates the capacitive response of the EDL. In our set-up, a 10 mV AC potential is swept across the electrode over the Nyquist frequency range per every immobilization step, and based upon the output response of our system, a capacitive impedance spectrum is built.

To validate the stability of the sensor response across the physiological pH of sweat, non-faradaic EIS, as explained above, was performed in synthetic sweat at pH 4, 6, and 8. Fig. 3b shows the results for the pH study on the rigid non-porous substrate. A detailed explanation behind the pH dependent response is further described in Section 2.4.3.

#### 2.3.3 Sensing response in human sweat

Once stability had been shown across the varying pHs, the immunoassay was repeated to build a Calibrated Dose Response (CDR). Human sweat contains a plethora of charged molecules and substances such as other amino acids, hormones, ions and even some other neuropeptides^27,28^ which can hinder or compound the signal response of the binding of NPY. While building a profile in synthetic sweat is helpful in the calibration of the sensor across the range of pH in sweat, building a dose response curve in actual human sweat with all its compounding constituents will validate the sensitivity and robustness of our sensor to the detection of NPY amidst the plethora of analytes present in human sweat. The CDR built an NPY profile in sweat by extracting the capacitive impendence (*Z*_imag_) after each dose immobilization step at 79 Hz which was where we observed the maximum capacitive response for the non-porous substrate. Doses ranged from 0.2 pg mL^−1^ to 100 pg mL^−1^ which covers the relative concentration of NPY in sweat as reported in literature^29^ for major depression disorder. For each dose concentration, the *Z*_imag_ is compared to a baseline *Z*_imag_ measurement which forms the *y*-axis of the curve shown in Fig. 3c. Based on the correlation between the ratio of change of *Z*_imag_ and the doses in pg mL^−1^, we performed regression analysis; non-linear in this case to get our CDR curve with an *R*^2^ value of 0.9666 indicative of the sensor's ability to detect NPY amidst the plethora of molecules present in sweat. We achieved an LOD of 0.2 pg mL^−1^ and a dynamic range of 0.2 pg mL^−1^ to 100 pg mL^−1^. The equation for the best line fit is also displayed in the graph in Fig. 3c. This equation can be leveraged to calculate the concentration of NPY in sweat based on the ratio of *Z*_imag_ to a baseline *Z*_imag_ measurement.

To test the efficacy of the CDR generated from our sensor, we performed a recovery experiment to see how well our CDR would be able to measure the concentration of sweat in pooled human sweat. We spiked pooled human sweat with NPY (10, 40 and 90 pg mL^−1^) and evaluated the immunoassay binding response on the sensor. Ratio off changes in *Z*_imag_ were performed in the same way as in the development of the CDR. Based on change of *Z*_imag_ calculated (*y*-axis), the *x*-axis concentrations of NPY was solved for from the fitting line equation of the CDR. Percentage recovered was calculated as
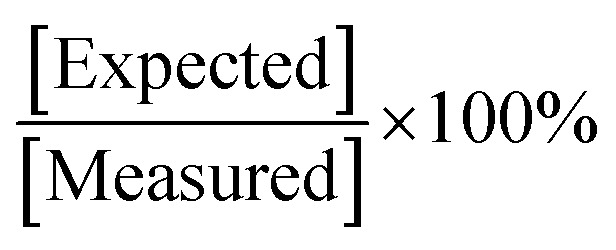


In analytical chemistry, recovery percentages between 70% to 120% of the expected value are deemed acceptable.^30^ The results of the recovery are shown in the graph in Fig. 3d. As clearly observed, the low, mid, and high doses fall in the desired percentage brackets as expected in recovery experiments. This clearly demonstrates the sensitivity of the sensor to build a robust CDR curve that can be used in the estimation of NPY concentrations in sweat. The Nyquist plot illustrated as Fig. S3.† The Nyquist plot shows the distribution of the *Z*_img_ component against the *Z*_real_ component of the impedance response. From the Nyquist, a dose dependent response can be observed. Using this plot, an equivalent electrochemical circuit fitting was performed to capture the binding response related modulations of the Randle's circuit elements. This electrochemical fitting of sensor response using a modified Randle's circuit is presented as Section S1.2.† From the fitting data, it can be observed that the response is driven by affinity binding, due to the dose dependant changes in the capacitive EDL component (modelled using a Constant Phase Element (CPE)) and *R*_ct_ component. With the increase in concentration of NPY in the system, the overall capacitance of the EDL increases due to increased interaction between the capture probe and target molecule. Being a protein, NPY is insulative in nature and upon binding on the surface of the electrode, it creates additional resistance to the charge flow at the electrode–electrolyte interface. This is characterized by the sudden increase in *R*_ct_ at higher dose concentrations of NPY (50–100 pg mL^−1^). Also, from the fitting data, it can be observed that the bulk solution resistance contributed by the bulk ions in human sweat does not significantly affect the response. Thus, the sensor response is directly correlated to the NPY binding events occurring at the capture probe/electrode–electrode interface.

The performance of the rigid non-porous substrate thus far is indicative of the use of our platform in the detection of NPY and the translatability to porous flexible substrates. Faradaic measurements were also taken on a non-porous substrate in the characterization of our immunoassay's response to NPY. The details and results are available in the ESI.†

### 2.4 Sensor performance in porous substrate

#### 2.4.1 Sensitivity using nano-porous substrates

**Fig. 4:**
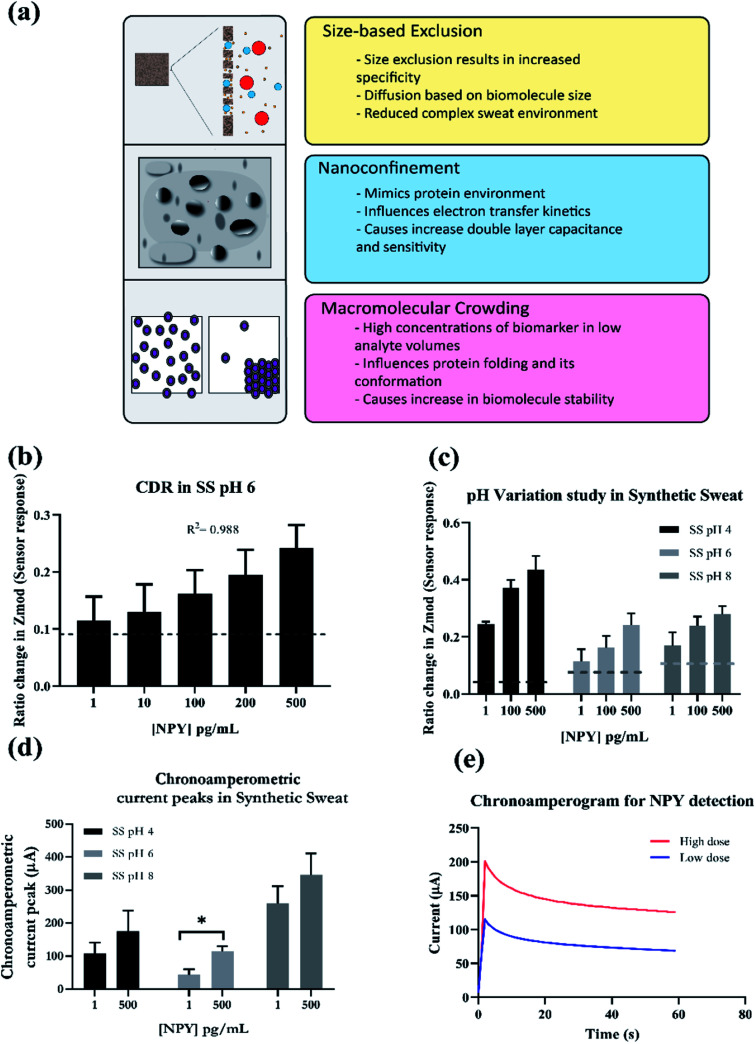
(a) Phenomena behind increased sensitivity for nanoporous membranes-size based exclusion, nanoconfinement and macromolecular crowding, (b) CDR response for NPY detection on porous platform in synthetic sweat pH 6, (c) pH variation study for NPY detection, (d) chronoamperometric current peaks (extracted) for low and high dose of NPY in synthetic sweat with varying pH, and (e) chronoamperogram for low and high dose of NPY in synthetic sweat.

One of the major hurdles in sweat-based sensing is making sure that the sensor is able to sensitively detect the target biomarker in concentrations at least a magnitude lower that those present in blood. This is true especially for NPY detection because the concentrations are in the picogram to lower nanogram range. Another challenge is detecting and producing a significant signal for slight changes in concentration. This work addresses those challenges by employing nanoporous membrane system for detection. Nanoporous membranes add to the detection efficiency of the system by multiple processes. The three main principles by which these membranes enhance sensitivity are macromolecular crowding, nanoconfinement and size-based molecular exclusion. A figurative explanation of these phenomena is provided in Fig. 4a. The contribution of macromolecular crowding towards signal enhancement is that higher amounts of biomarkers are concentrated/packed inside the membrane. This leads to prolonged interactions between the target and receptor, thus enhancing the binding. Crowding also increases the biomolecule stability in the system, which is highly advantageous while using proteins as target and receptor.^31^ Nanoconfinement helps by influencing the charge transfer kinetics and increasing the surface area of interaction, which in turn increases the double layer capacitance. This specially benefits assays which rely on non-faradaic capacitive modulations for detection of a binding response,^15,32^ such as the one used in this system. Lastly, size-based exclusion provides diffusion driven, selective molecular transport across the membrane. This helps when detection is performed in highly ionic solutions like sweat. It helps reduce the non-specific binding interactions and increases specificity of the reaction.^22^

#### 2.4.2 Sensor detection response using electrochemical impedance spectroscopy (EIS)

Electrochemical impedance spectroscopy is widely used as a label-free sensing modality.^33^ For sweat based sensing, EIS offers good signal resolution, rapid sensing, and facilitates ease of operation from a monitoring device perspective.^33^ Using EIS for capturing the sensitivity of the response has many advantages. First, EIS helps in capturing the real time interfacial changes in electrical double (EDL) due to target binding. Secondly, with rapid transduction and processing capability, EIS can perform fast detection, making it ideal for a time-sensitive biomarker tracking system. Finally, by using non-faradaic EIS, the system can be made label free which improves the specificity and economical value of the sensing system. In the previous section, performance of the sensing response using EIS was demonstrated on a non-porous platform. By using a porous substrate, molecular transport is more effective and interaction increases, elevating the overall capacitance of the system. As discussed in the Introduction, at physiological pH conditions *i.e.* pH 6–7, the net charge of NPY molecule ranges from +0.2 to +1.^34^ This charge, combined with nanoporosity of the substrate, helps in amplifying the sensing response of the system, making detection without a redox probe equally as sensitive as faradaic sensing. The binding response between the target and the immobilized assay is illustrated in Fig. 4. The result of the sensor's response with respect to increments of NPY concentration is presented in Fig. 4b. This represents extracted impedance data at 100 Hz, where we see the maximum capacitance behaviour for the entire system. The ratio of change formula is provided in the previous publication.^35^ The concentrations tested range from 1 to 500 pg mL^−1^ prepared in a sweat analogue solution with pH 6. This is to gauge the sensor response in physiologically stable sweat conditions. According the results, the sensor shows a good correlation to the NPY concentration in the system. The impedance response changes increase proportionally to the changes in concentration. The mechanism behind this dose response change is the affinity binding phenomenon occurring at the EDL interface. The Self Assembled Monolayer (SAM), which is immobilized on the sensor surface is responsible for contributing towards the detection signal. The changes in target concentration in the system are reflected as capacitive modulations in the electrical double layer. Non-faradaic EIS works on the principle of tracking the changes in the capacitance of the electrical double layer within a certain frequency range.^36^ Usually, this range lies between 1–1000 Hz as this corresponds to the perturbation at the interface, whereas higher frequencies correspond to perturbation at a bulk solution level.^26^ The changes in impedance suggest that the system is sensitive to the amount of NPY present. The data shows a correlation of *R*^2^ of 0.98, which indicates that the sensor is sensitive to the NPY concentration increments in the system. The limit of detection of the sensing system is 10 pg mL^−1^ with a dynamic range between 10 and 500 pg mL^−1^. This is relevant to the physiological levels of NPY which is 50–200 pg mL^−1^. This indicates that at physiological levels, the sensor will be able to detect the presence of NPY in the system even for the picogram range. This is of prime importance to a system used for self-monitoring. For anxiety monitoring, real time binding interactions provide useful information about the spike and fall of the anxiety inducing biomarker levels, in this case, NPY levels.

#### 2.4.3 Sensor detection response in presence of pH variation

The charge status of NPY is influenced by the pH of the buffer. With the increase in pH, the net charge of NPY is reduced due to the interactions with ions from the solution. This is due to the increased electrostatic interactions between the positively charged amino acid residues and the ions present in the buffer.^37^ As discussed earlier sweat pH fluctuates between 4 and 8, driven by the biophysiological processes occurring in the body.^38^ In the previous section, it was demonstrated that the non-porous platform was able to differentiate between the low and high levels of biomarker in the presence of pH fluctuations. In this section, we evaluated the effect of pH on sensor response. This is illustrated in Fig. 4c, the sensor response for low, medium, and high dose concentration was tested in synthetic sweat. It can be observed from the data that the sensor's sensitivity to NPY levels is conserved when exposed to pH 4, 6, and 8. A similar trend is seen when observing the non-porous system, with highest sensor response observed at pH 4, followed by pH 6 and 8. The isoelectric point (pI) of NPY lies at 7.9.^39^ In the presence of an acidic pH, the basic residues of NPY dominate the net charge. For pH 4, the sensor response increased by a magnitude of 20% with each concentration increase. The response might be because of several hypotheses. The NPY residues are in a monomeric state, with this the binding to the receptor *i.e.* NPY antibody is linear and the overall charge displacement is higher. This displacement creates significant changes in the EDL capacitance captured as impedance change. With the increase in pH, the structure converts to a dimeric form and as it moves towards its isoelectric point causing the net charge to shift towards zero.^40^ This is evident by the decrease in range of sensor response between low and high doses for pHs 6 and 8; however, there is still a significant increase in the impedance response to the NPY concentration. Also, the sensing trend of the detection system is conserved, with similar sensitivity to the different concentrations of NPY, despite the pH variations. This indicates that the system conserves the NPY related binding sensitivity in the presence of pH fluctuations. Comparing it to the non-porous system pH variation response, the signal resolution has increased for the porous system. A hypothesis for this increase is the increase in surface area of interaction and the porosity dictated unfolding and folding of the protein leading to enhanced interactions between molecule and receptor.^41^

#### 2.4.4 Sensor response using chronoamperometry

To test the robustness of the system, sensor performance under an alternate electrochemical technique was evaluated. Typically, a chronoamperometry experiment involves applying a step potential close to the redox potential of the target molecule and monitoring the rate of change of output current response. This DC based technique was used to evaluate and to reject the hypothesis that experimental perturbation is responsible for response change instead of binding response. This detection technique is useful for investigating the effects of diffusion and determining the kinetics of the reaction. The advantage of using this electroanalytical technique over traditional techniques like Cyclic Voltammetry (CV) is all of the abovementioned reaction information is captured in a single experiment and is in real-time.^26^ Another advantage of using chronoamperometry is that it facilitates off-site/non-laboratory setting based detection using small sample volumes.^42^ A disadvantage of this technique is that the effects observed are often bulk changes in the testing medium instead of capturing specifically the electrode–electrolyte interfacial related changes or EDL based capacitive modulations. A comparative analysis of different chronoamperometric studies and their experimental details has been provided in Table 3. This chronoamperometry study was carried out with synthetic sweat with different pH, to characterize the pH driven response of the sensing platform. The results are illustrated in Fig. 4d. A chronoamperogram consists of a sudden current spike in response to the step potential applied, followed by an exponential decay in the current. This current then stabilizes to steady-state current. The current peaks were extracted to characterize the dose dependant response of the sensing system in the presence of low and high doses of NPY. The change in peak current ranges from 107 ± 33 μA to 175 ± 62 μA for pH 4, followed by 43 ± 16 μA to 114 ± 16 μA for pH 6, and 259 ± 52 μA to 346 ± 64 μA for pH 8. From the results, it can be observed that there is a significant change between the response for low and high dose of NPY for pH 4 and 6. The trend remains similar for pH 8, where the response increases with increases in concentration. This behaviour can be explained by the hypothesis that the charge drives the NPY behaviour and affects the perturbation response. However, the sensor is still able to detect and respond to the changes in NPY concentration. During the chronoamperometry measurements, the system is perturbed by a DC potential of 600 mV (according to its redox potential^48^). This perturbation is applied at the working electrode against the reference electrode. The working electrode is where the target binding reaction occurs and the counter electrode acts as a sink for the current necessary to maintain the potential at the working electrode. The capacitive modulations here are a response to the bulk and interfacial changes due to target binding. For the potential applied at pH 4 and 6, the net charge on NPY is positive, this causes electrostatic repulsions at the potential, which is reflected as the lower values for signal response. However, because of the enhanced interactions with the capture probe due to the monomeric state of the protein form, the signal resolution is high, with *p* value < 0.05. Similarly, at pH 8, at the isoelectric point of the protein, net charge is zero and protein adopts a dimeric complex form, due to which the input perturbation results in a higher signal response. This could also be accompanied by charge screening effects observed at the alkaline pH.^35^ It can be inferred from these results that using an alternate technique confirms the sensitivity of the system to the NPY concentration in the system. Also, combining the detection immunoreceptors with a nanoporous substrate, the sensing capability is conserved in the presence of pH fluctuations.

**Table 3:** Chronoamperometric detection modality-based biosensors

Research	Target, buffer	Detection limit	Experimental	Reference
Disposable screen-printed amperometric biosensor utilizing monoclonal antibody as detection probe	Progesterone, phenol solution, cattle milk samples	1 × 10^−9^ mol dm^−3^, 5 ng mL^−1^	+0.70 V *vs.* saturated calomel (SCE)	42 and 43
Immunosensor development using polypyrrole-based layer for entrapping bovine leukemia virus (BLV) protein using antibodies	gp51 protein (BLV antigen), bovine serum	10-Fold dilutions of serum, antigen–antibody complex driven response, concentrations not mentioned	0 to 600 mV *vs.* Ag/AgCl, potential pulses of +950 mV *vs.* Ag/AgCl	44
Gold nanoparticle decorated polyaniline nanowire for covalent attachment of biorecognition elements – glucose oxidase enzyme (GOx), ssDNA, and lamin A antibody	Glucose, complementary DNA strand, and lamin A protein, phosphate buffer solution	1 μM for glucose (chronoamperometric detection)	Operating potentials of −0.1 V and +0.8 mV	45
Cellular biosensor for detection of aflatoxin B_1_ (AFB_1_) using screen-printed electrodes (SPEs) and anti AFB_1_ antibody as biorecognition element, and engineered/plain monkey vero kidney cells	Aflatoxin B_1_ (AFB_1_)-mycotoxin, phosphate-buffered saline	0.05 ng mL^−1^ (plain cells) and 1.5 ng mL^−1^ (vero-anti-AFB1 cells)	−100 mV potential	46
Chronoamperometric immunosensor using anti-TNF-α antibody functionalized on gold electrode using 4-carboxymethylaniline (CMA)	Tumor necrosis factor (TNF) – α artificial saliva, saliva	1 pg mL^−1^	100 mV *versus* the saturated calomel reference electrode (SCE)	47

### 2.5 Sensor specificity

**Fig. 5:**
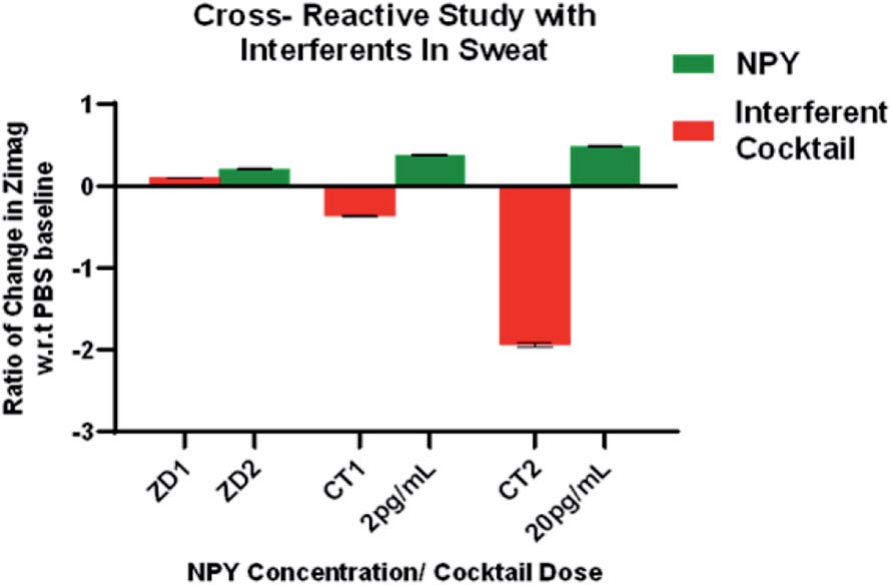
Cross-reactivity study with interferents.

Detection of sweat-based protein biomarker at low concentrations *via* electrochemical modalities is challenging because of the highly ionic composition of sweat. Issues can arise due to non-specific binding from components like uric acid lactate, creatinine, and ascorbic acid, and glucose.^49^ A synthetic analogue representing the physiological concentrations of these interferent molecules was created. This formulation was used to characterize the non-specific response of the sensing platform. The response for NPY was compared with the dosing of interferent solution on the sensor interface. Fig. 5 illustrates the specific response for NPY and shows significant changes from baseline for NPY concentrations of 2 pg mL^−1^ and 20 pg mL^−1^. The non-specific response does not follow a specific trend and the results indicate a negative change in impedance, indicating that the presence of interferents does not affect the immunoassay and produce false positives. The response for specific NPY concentrations is significantly higher than the interferent buffer dosing with *p* < 0.05. The specificity of the response is triggered by the interfacial responses caused by anchoring of the protein on the surface of the electrode creating capacitive modulations. The testing scenario is presented for highest physiological conditions of interferents. However, in real wearable monitoring scenarios, the concentrations of interferents present would be at a lower level due to the resting, passive state of the user. These results conclude that the sensor response is specific for NPY and is suitable for monitoring in a wearable form factor *via* sweat.

### 2.6 Portable sensor electronics interface

As a wearable, a device of this nature needs to provide actionable, useful information to the end user. After collection, data sets must be analysed and categorized. One can use a simple thresholding system or a more complex classification system to categorize the data. In the case of our device, we would use the change in impedance or the change in the chronoamperometric peaks to classify the sample into a low, medium, or high concentration. With respect to impedance spectroscopy, the simplest way to do this would be to pick a specific operating frequency where the highest sensitivity is seen in the original dose responses. A gradient would then be established, and thresholds could be defined. Because there is some sensor to sensor and dosing variability, the baseline must be recorded and the percent change in impedance should be the metric used to classify the data. The same concept can be employed for chronoamperometric peaks currents. This method of classification makes the user experience straightforward. The final aim of this work is to run human subject studies in order to have a better understanding of sweat concentrations before and after specific stimulation events. This would involve either wearing one of the porous sensors to later be tested on a benchtop form factor device, wearing the device with the porous sensor attached for live measurements, or using pooled human sweat with a rigid electrode system.

**Fig. 6:**
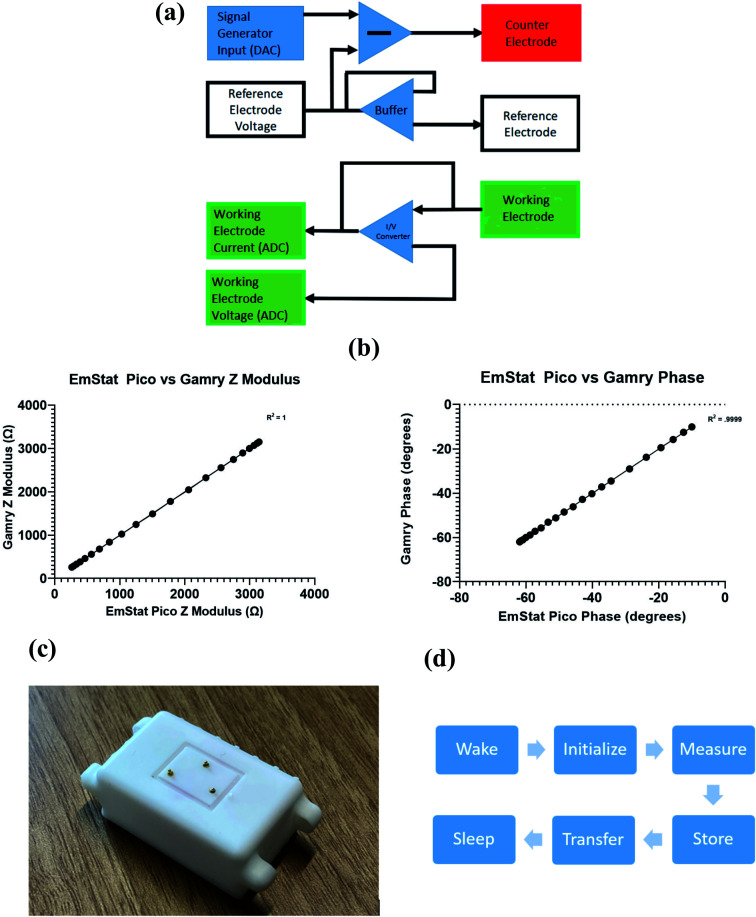
(a) Block diagram of ADuCM355, (b) correlation plots comparing the EmStat Pico to the Gamry response, (c) wearable form factor 3D printed housing with pogo pins, and (d) basic state machine logic implemented to improve battery life and wireless communication efficiency.

Two alternative electronics form factors were designed to suit the final application of this work. These would also help validate the findings through the standard benchtop electrochemical instrumentation such as a Gamry potentiostat. We opted to use the EmStat Pico, which is a small form factor potentiostat. This features the ADuCM355 microcontroller with an onboard potentiostat. The process flow is illustrated in Fig. 6a. This device would then be paired with our rigid substrate electrode system and the porous electrode system to provide human sweat results. This system was designed to avoid significant physical stimulation so that we could have a better understanding of resting neuropeptide-Y concentrations.

The benchtop form factor requires the implementation of the UMFT234XD USB FTDI adaptor. This effectively turns the EmStat Pico into a thumb drive size device capable of taking measurements. Neuropeptide-Y has a very low charge at physiological pH; hence, the impedance, phase, and current changes will be quite small and require high resolution hardware. To assess the benchtop configuration's stability and accuracy, a controlled sweep from 10 to 1000 Hz on both the benchtop form factor and the Gamry potentiostat was performed. From Fig. 6b, a strong correlation between the two data sets (*p* > 0.99) was observed. This confirmed that the device would be viable for validation and further testing. It also ensures that the operating frequencies of our EIS scans will be stable.

The mobilized form factor would involve the addition of a voltage regulator and a 5 volt battery to power the system. A bluetooth module would also need to be added to the design so that this form factor could pair with the PSTouch Android software provided with the EmStat Pico. We would then use a gold plated pogo-pin system to interface with the porous substrate-based electrode system. An image of this interface can be found in Fig. 6c.

The core limitations of this design are artifact generation and battery life. The major artifacts generated are from human motion, such as walking. The built in Hann window on the ADuCM355 suppresses the noise from external artifacts by creating a strict high and low pass system on either side of the chosen operating frequency. As for battery life, we would employ a state machine to limit the microcontroller's on time by utilizing its sleep function. Additionally, data can be stored on the microcontroller's onboard flash memory and sent in bulk to limit the bluetooth module's on time. The general process flow of such state machine can be seen in Fig. 6d.

## 3 Experimental

### 3.1 Reagents and materials

NPY antibody and protein were purchased from Abcam (Cambridge, MA, USA). The thiol linker, DSP (Dithiobis-[Succinimidyl Propionate]) was procured from Sigma-Aldrich (St. Louis, MO, USA). The solvent, DMSO (Dimethyl Sulfoxide) and PBS (Phosphate Buffered Saline) were obtained from Thermofisher Scientific Inc. (Waltham, MA, USA). Sweat analogue used were prepared according to the protocol described in previous publication.^35^ Milipore DI water was employed for making dilutions and buffers. Pooled human sweat was purchased from Lee Biosolutions Inc. (St. Louis, MO, USA).

### 3.2 Fabrication of sensing platforms

Non-porous electrode substrates were procured from PCB Universe Inc (Vancouver, WA, USA). Porous gold sensors were fabricated on flexible nanoporous polyamide substrate (GE Healthcare Life sciences (Piscataway, NJ, USA)) using a shadow mask technique. Gold was deposited using thin film deposition using a Temescal E-beam evaporator available in the cleanroom facility provided by the University of Texas at Dallas. The functionalization protocol is described in previous literature.^50^ Here the NPY antibody concentration used was 10 μg mL^−1^.

### 3.3 Experimental design for FTIR

FTIR spectrum was captured using Thermo Scientific Nicolet iS50 FTIR in Attenuated Total Reflectance (ATR) mode. The instrument consists of a germanium ATR crystal, deuterated triglycine sulfate (DTGS) detector and a KBr window. Samples for analysis were prepped on a glass substrate with washing and drying steps in between immobilizations to remove physisorbed molecules. The spectrum was recorded between 800 cm^−1^ and 4000 cm^−1^ with a resolution of 0.5 cm^−1^ and 256 scans.

### 3.4 Experimental design for electrochemical impedance spectroscopy

Following functionalization, synthetic sweat (5 μL) spiked with NPY concentrations was dispensed onto the sensor surface. An AC potential bias of 10 mV was applied to the sensor and impedance spectra was recorded for the Nyquist frequency range (1 Hz to 1 MHz). This Calibrated Dose Response (CDR) study was performed using Gamry Reference 600 potentiostat (Gamry Instruments, PA, USA). All the impedance data is extracted at 100 Hz, where peak capacitive behaviour of sensing system was observed.

### 3.5 Experimental design for chronoamperometry measurements

For chronoamperometry measurements, a similar CDR protocol was used. DC step potential of 600 mV was applied to the electrode and current spectra was recorded for the period of 60 seconds. This was also performed using Gamry Reference 600 potentiostat (Gamry Instruments, PA, USA). The current peaks were extracted, and post analysis was performed using Echem Analyst™ software.

### 3.6 Experimental details for portable electronics

EmStat Pico, a small form factor potentiostat, was created by PalmSens and Analog Devices. This features the ADuCM355 microcontroller with an onboard potentiostat. Electrodes fabricated from Section 3, were paired with this system to collect sweat and perform detection. Different revisions of portable device design, one benchtop and one wearable, with a 3D printed design was created using the Formlabs Form 2 3D printer.

### 3.7 Statistics

Data is represented and plotted as mean ± SEM with *N* = 3. The sensor–sensor variability is under 20% which is CLSI guidelines compliant.^51^ Significance study between the groups was carried out using ANOVA followed by *post-hoc* Tukey test. For the significance study, which was carried out withing two groups, an unpaired two tailed *T*-test was performed. All the statistical studies, along with the correlation ad regression analysis presented in this work were performed using the statistical software Graph Pad Prism v8.01. (Graph Pad Software Inc., La Jolla, CA, USA).

## 4 Conclusions

The work demonstrates the development of a wearable chronic disease management platform for MDD and anxiety disorders. In this work, comparison of electrochemical detection response between non-porous and porous substrates for optimal sensing performance. Detailed electrochemical analysis of sensor performance using various detection techniques like faradaic, non-faradaic EIS and chronoamperometry is provided. Electrochemical fitting of sensor response using modified Randle's equivalent circuit highlighted the dose dependent capacitive sensor response of the system. Detection limit of the sensing system is 10 pg mL^−1^ and dynamic range of operation is 10–500 pg mL^−1^. The sensor response is conserved in presence of pH fluctuations. Non-specific binding study using interferents indicates that sensor is specific to NPY molecule. Finally, novel portable electronic system highlights the wearable aspect of the device and provides insight into further application. Overall, this novel wearable NPY biomarker tracking system is envisioned to be a chronic anxiety and MDD management platform *via* self-monitoring.

## Author contributions

Data collection, analysis, writing: NC, SU, PR, SB; conceptualization, writing, analysis, reviewing: SP.

## Conflicts of interest

Dr Shalini Prasad has a significant interest in Enlisense LLC, a company that may have a commercial interest in the results of this research and technology. The potential individual conflict of interest has been reviewed and managed by The University of Texas at Dallas and played no role in the study design; in the collection, analysis, and interpretation of data; in the writing of the report, or in the decision to submit the report for publication.

## Supplementary Material

RA-010-D0RA03729J-s001
