# Supplementary material for: Development of a flexible, sweat-based neuropeptide Y detection platform
Source: RSC Adv. 2020 Jun 17;10(39):23173–86. doi: 10.1039/d0ra03729j (PMC9054693; doi:10.1039/d0ra03729j)
Supplement: RA-010-D0RA03729J-s001 [file RA-010-D0RA03729J-s001.pdf]

Electronic Supplementary Material (ESI) for RSC Advances.
This journal is © The Royal Society of Chemistry 2020

Supplemental information

Development of flexible, sweat-based NPY detection platform

Nathan Churcher[†a], Sayali Upasham[†a], Paul Rice [a], Serena Bhadsavle [a], Shalini Prasad*[a]

S1.1: Faradaic sensor response in synthetic sweat:

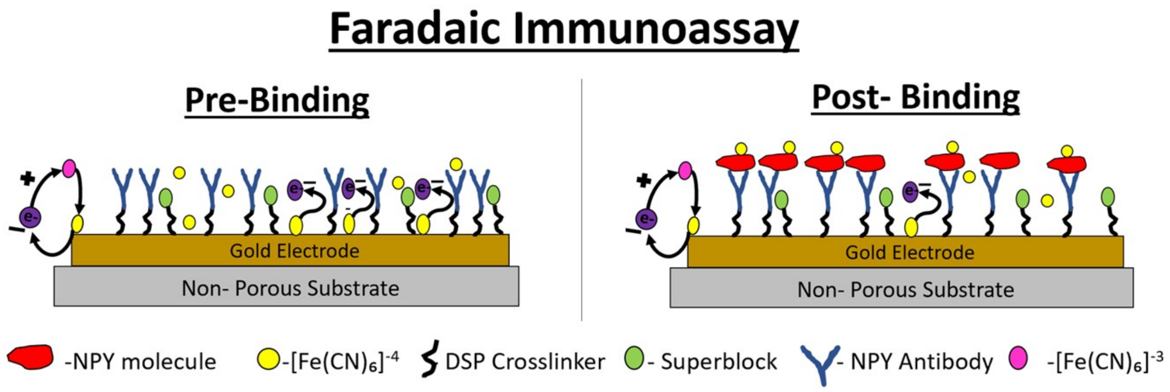

Figure S1: Faradaic immunoassay schematic

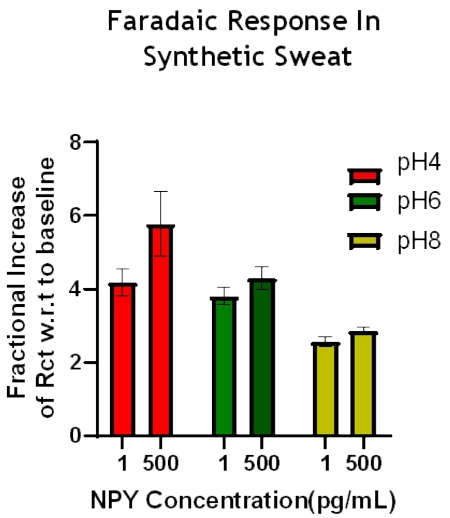

Figure S2: Faradaic sensor response is synthetic sweat

S1.1: Faradaic sensor response in synthetic sweat: In the characterization of the immunoassay, we employed the use of Faradaic EIS, an altered form of the Non- Faradaic process described above. In Faradaic EIS, we introduce a redox mediator, $K_3[Fe(CN)_6]$ to increase the charge transfer capabilities of the system. While in Non- Faradaic EIS, the capacitive changes in the EDL are characterized, in Faradaic

EIS, the focus is the characterization of the resistance to charge transfer (Rct) at the electrode- electrolyte interface. As shown in the schematic below, (Suppl. A) we are interested in characterizing the changes in Rct as more and more binding occurs. Suppl. B shows the results of the pH study with Faradaic EIS, the discussion to the results are expanded on in section 2.4.3 above.

S1.2: Electrochemical fitting for Non-faradaic sensor response:

Table S1: Theoretical Fit of Experimental Results for the capacitive and resistive components of the Equivalent Modified Randle's Circuit

Spiked Concentration(pg/mL)	R_s(Ω)	EDL-T(μF)	EDL-P (Coefficient -n)	R_{ct} (Ω)
**0**	81.98	5.9479	0.88129	189390
**0.2**	28.65	7.141	0.87618	193020
**2**	27.84	8.1328	0.86098	201140
**20**	26.44	9.1452	0.85298	183580
**50**	26.87	10.06	0.84178	289540
**100**	25.66	10.766	0.83807	466840

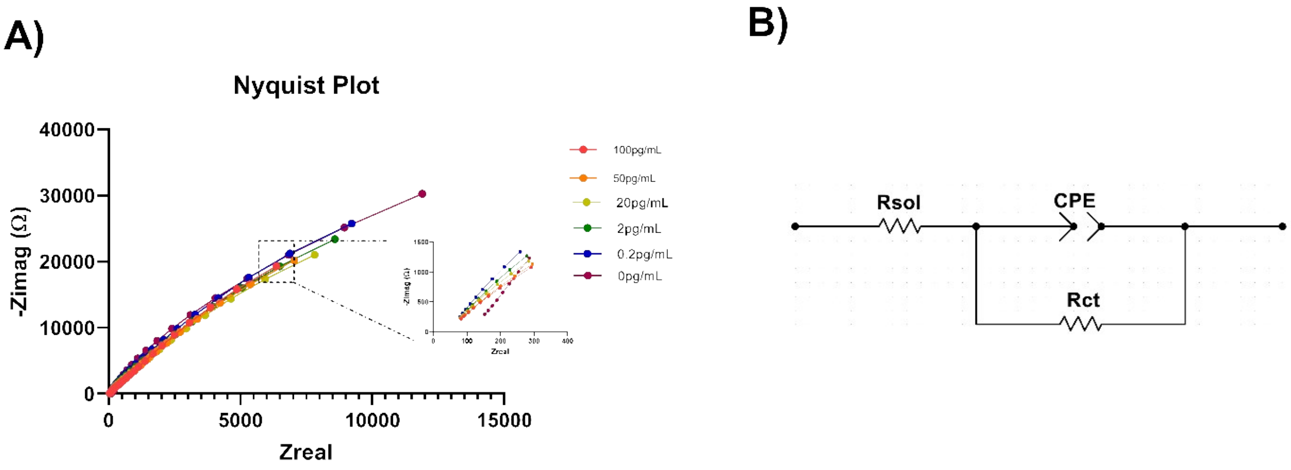

Figure S3: A) Nyquist showing the dose NPY dose response. B) Equivalent Modelled Randle's circuit used to model the electrochemical interactions occurring at the electrode- electrolyte interface.

S1.2: Electrochemical fitting for non-faradaic sensor response: The interactions occurring at the electrode- electrolyte surface were modelled using the modified Randle's circuit as shown in Fig S3. The impedance due to the bulk of the solution was modelled with a resistor (Rsol), whereas the impedance arising to the transfer of charges was modelled with a resistor (Rct). The electrical double layer formed was modelled with a constant phase element (CPE). EDL-P from Table S1 shows the phase of the CPE, the closer the value of EDL-P is to 1, the more capacitive the response is which supports our hypothesis of the formation of a capacitive double layer at the electrode-electrolyte interface which is modulated with increasing binding of NPY. The relatively higher impedance observed from the Rct and suppressed semi-circle buttress the capacitive response of our system.
